# ^18^F-FDG PET/CT features of Meigs syndrome induced by ovarian sex cord stromal tumors: a retrospective clinical study

**DOI:** 10.1038/s41598-024-51186-5

**Published:** 2024-01-03

**Authors:** Xianwen Hu, Wenxin Li, Xiaotian Li, Dandan Li, Jiong Cai, Pan Wang

**Affiliations:** 1 Department of Nuclear Medicine, Affiliated Hospital of Zunyi Medical University, 149 Dalian Road, Huichuan District, Zunyi, 563000 China; 2 Department of Obstetrics, Zunyi Hospital of Traditional Chinese Medicine, Zunyi, 563000 China

**Keywords:** Diseases, Medical research, Oncology

## Abstract

The objective of this study was retrospectively to analyze the clinical characteristics and ^18^F-FDG PET/CT findings in Meigs syndrome (MS) patients. A total of 21 patients with MS induced by ovarian stromal tumors and 69 patients with pseudo-MS caused by ovarian cancer (OC-PMS) were subjected to evaluation using ^18^F-FDG PET/CT. Visual and semi-quantitative methods were employed to analyze the PET/CT findings. Visual analysis included recording whether the density of the primary tumor was uniform, whether there were cystic changes and calcifications, and the location of serous fluid accumulation. Semi-quantitative analysis involved the measurement of the tumor size, SUVmax, and SUVmean. No significant difference was observed in the size and density of primary tumors between the MS group and the OC-PMS group. However, the SUVmax and SUVmean of tumors in the MS group were found to be significantly lower than those in the OC-PMS group. The amount of serous cavity effusion caused by ovarian sex cord stromal tumors was found to be unrelated to the size of the tumor, SUVmax, and SUVmean but was positively correlated with the level of Ca125. MS patients have both benign ovarian tumors and ascites and/or pleural effusion, which may be accompanied by elevated Ca125 levels. This should be considered as one of the differential diagnoses for ovarian cancer. Understanding the PET/CT features of MS can facilitate the attainment of an accurate diagnosis before surgery.

## Introduction

Meigs’ syndrome (MS) is characterized by the presence of benign ovarian tumors accompanied by ascites and/or pleural effusion, which spontaneously resolve upon the removal of the ovarian tumor. It is frequently associated with sex cord stromal tumors, including ovarian fibroma, thecoma, theca fibroma, and granulosa cell tumor, with a higher prevalence in postmenopausal women, typically occurring around the age of 50^1,2^. In addition, other pelvic masses can also cause pleural effusion or ascites, which is called pseudo Meigs syndrome, including benign pseudo Meigs syndrome caused by benign tumors outside the ovary or broad ligament, and malignant pseudo Meigs syndrome caused by ovarian malignant tumors^3^. Clinical manifestations of MS are non-specific, often manifesting as dyspnea, abdominal distension, abdominal pain, fatigue, and weight loss^4^. The exact mechanism behind serous cavity effusion in MS remains unclear. Ascites may result from direct tumor secretion, lymphatic obstruction due to compression and peritoneal stimulation, while pleural effusion formation may be attributed to the translocation of ascites through the lymphatic or diaphragmatic routes into the pleural cavity^5^. Additionally, it has been reported that the development of pleural effusion and ascites may be linked to the release of inflammatory cytokines and growth factors, such as vascular endothelial growth factor, fibroblast growth factor, interleukin 6, and IL-8, which affect the pleura, peritoneum, and pericardium, leading to increased exudation. Following tumor resection, the disappearance of serous cavity effusion is accompanied by a decrease in the levels of these cytokines in the serum^6,7^. Patients with MS who exhibit elevated serum Ca125 levels are often misdiagnosed as having ovarian epithelial malignancies^8^. The objective of this study is to retrospectively analyze the clinical characteristics and ^18^F-FDG PET/CT findings of MS to enhance our understanding of the condition and achieve an accurate preoperative diagnosis.

## Patients and methods

This retrospective study received approval from the institutional review board of Zunyi Medical University’s affiliated hospital, and all methods were performed in accordance with the relevant guidelines and regulations.

### Patients

A retrospective analysis was carried out on patients who were admitted to the Affiliated Hospital of Zunyi Medical University between January 1, 2019, and July 30, 2023. These patients were histologically confirmed to have ovarian sex cord stromal tumors, including fibroma, thecoma, theca fibroma, and granulosa cell tumor, and were accompanied by ascites and/or pleural effusion. Additionally, patients with pseudo-Meigs syndrome induced by ovarian cancer (OC-PMS) were included as control subjects. PET/CT imaging features and clinical data were extracted. Inclusion criteria encompassed patients with a pathological diagnosis of ovarian sex cord stromal tumors or ovarian cancer, combined with ascites or pleural effusion, and the subsequent disappearance of pleural and ascitic fluid within two weeks following surgical tumor removal. Exclusion criteria included: (i) instances where the image quality was insufficient for accurate analysis and (ii) situations where the lesion area had been subjected to any prior treatment, such as surgical resection, before the ^18^F-FDG PET/CT examinations.

#### ^18^F-FDG PET/CT

^18^F-FDG was produced and synthesized using the F300E modular automatic synthesis unit of the HM-10HP cyclotron FDG chemical synthesis system (Sumitomo, Japan). Imaging was performed when the required radiochemical purity of ^18^F-FDG exceeded 95% after intravenous injection. Prior to the examination, patients were instructed to fast for at least 6 h, and fasting blood glucose levels were controlled to be below 11.1 mmol/L. Imaging was conducted 1 h after the injection of ^18^F-FDG (0.1–0.15 mCi/kg) using a Biograph mCT PET/CT scanner (Siemens, Germany). The scanning range extended from the top of the skull to the middle of the femur. CT scanning parameters included a tube voltage of 120 kV, tube current of 119 mA, and a slice thickness of 5 mm. PET scanning was immediately performed after the completion of CT, with scanning parameters of 2 min per bed and a total of 6 to 7 beds. PET images were corrected for attenuation using CT data and reconstructed using the TrueX + TOF method following image acquisition.

### Image analysis

Visual and semi-quantitative analysis of PET/CT images were carried out by two nuclear medicine diagnosticians, each having a minimum of 5 years of experience. Visual analysis involved the recording of the location of fluid in the serous cavity (pleural effusion, ascites, or both) and the assessment of the density of primary ovarian tumors (uniform or non-uniform, presence of cystic changes, and calcifications). For semi-quantitative analysis, a region of interest (ROI) was placed over the entire lesion area, and the maximum standardized uptake value (SUVmax), mean standardized uptake value (SUVmean), and the size of the primary ovarian tumors were calculated for evaluation purposes. The amount of fluid accumulation in the serosal cavity is determined by measuring the vertical distance between the visceral and parietal serosal membranes at the location with the highest amount of fluid accumulation.

### Statistical methods

The statistical analysis was conducted using R language (version 4.3.1). For continuous variables, normality was initially assessed using the Shapiro–Wilk test. If the data followed a normal distribution, they were presented as mean ± standard deviation, and comparisons among multiple groups were carried out using one-way ANOVA. In cases where the data did not exhibit a normal distribution, statistical description was presented as the median (Q1, Q3), and between-group comparisons were performed using the independent samples rank sum test. For count categorical data, the presentation consisted of the number of cases (%) and between-group comparisons were conducted using the chi-square test. If the conditions for the chi-square test were not met, Fisher’s exact probability method was applied. Correlation analysis was executed using Spearman’s method and the results were visualized through scatter plots. All tests were two-sided, and statistical significance was considered when *P* < 0.05.

### Ethics approval and consent to participate

This retrospective study approved by the ethics committee of the affiliated hospital of Zunyi medical university. Informed consent was waived due to retrospective nature of study by ethics committee of the affiliated hospital of Zunyi medical university.

## Results

### Clinical and ^18^F-FDG PET/CT features of patients with MS versus OC-PMS

**Figure 1:**
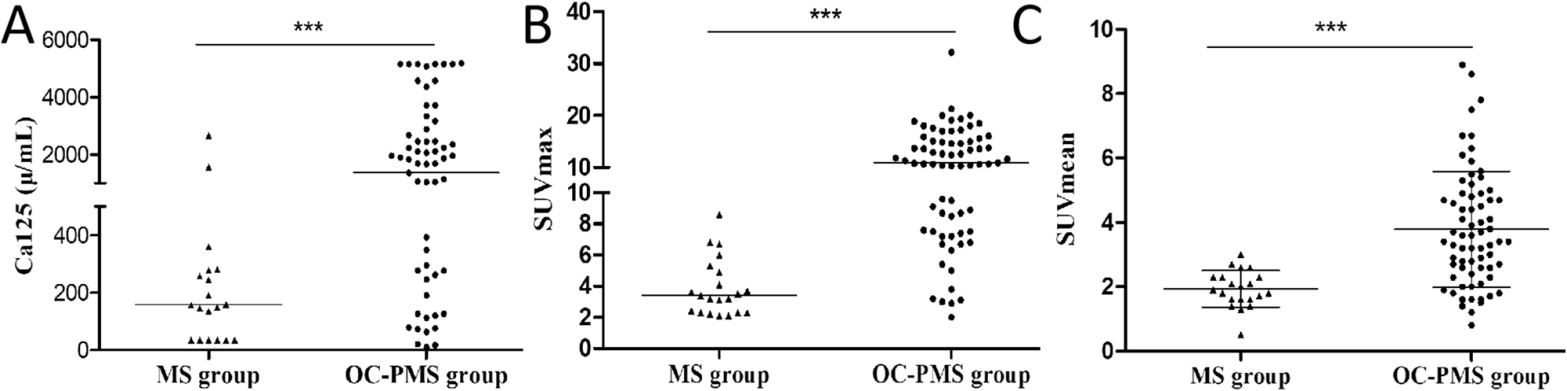
The serum Ca125, SUVmax and SUVmean of ^18^F-FDG PET/CT imaging in patients with MS and pseudo-meigs syndrome due to ovarian cancer (OC-PMS). (**A**) The serum Ca125 in the MS group compared with those in the OC-PMS group; (**B**) SUVmax in the MS group versus OC-PMS group; (**C**) SUVmean in the MS group versus OC-PMS group. Notes: MS group (n = 21), OC-PMS (n = 69); ***P < 0.001.

A total of 21 patients afflicted with MS induced by sex cord stromal tumors and 69 cases of OC-PMS underwent assessment via ^18^F-FDG PET/CT. The clinical features and PET/CT findings are summarized in Supplementary file. Both MS and OC-PMS groups exhibited nonspecific clinical symptoms, including abdominal pain, bloating, weight loss, chest pain, chest discomfort, and difficulty breathing. There was no significant difference in age, primary tumor size, or tumor density in CT scans between the MS and OC-PMS groups (as shown in Table 1). However, the levels of serum Ca125, as well as the SUVmax and SUVmean of the primary tumor on PET, displayed significant disparities between the MS group and the OC-PMS group, with the latter exhibiting notably higher values (as illustrated in Fig. 1). Furthermore, apart from one patient with pericardial effusion complicated by inflammation, displaying ^18^F-FDG uptake, no increase in ^18^F-FDG uptake was observed in serous cavity effusion among the MS group. Conversely, in the OC-PMS group, serous cavity effusion was predominantly associated with focal and diffuse increases in ^18^F-FDG uptake.

**Table 1:** Clinical and PET/CT features of patients with MS versus OC-PMS.

Parameters	MS group (N = 21)	OC-PMS group (N = 69)	*P*
Age (years)	54.33 ± 13.71	55.70 ± 9.94	0.618
Ca125 (μ/mL)	174.85 [60.10; 279.95]	1364.00 [322.10; 2570.00]	< 0.001
PTS (cm)	9.31 ± 3.81	9.25 ± 4.45	0.963
Density			
Cystic dominance	3 (14.29%)	15 (21.74%)	0.455
Solid dominance	18 (85.71%)	54 (78.26%)	
Cystic change			0.052
No	7 (33.33%)	5 (7.25%)	
Yes	14 (66.67%)	64(92.75%)	
Calcification			0.608
No	14 (66.67%)	50 (72.46%)	
Yes	7 (33.33%)	19 (27.54%)	
SUVmax-primary tumor	3.40 [2.30; 5.10]	10.60 [6.75; 15.45]	< 0.001
SUVmean-primary tumor	1.93 ± 0.12	3.79 ± 0.22	< 0.001
SUVmax-SCE	1.10 [0.70; 6.38]	6.79 ± 2.93	< 0.001
SUVmean-SCE	1.05 [0.73; 3.20]	3.07 ± 1.45	< 0.001

*MS* Meigs syndrome; *OC-PMS* Pseudo-meigs syndrome due to ovarian cancer; *SCE* Serous cavity effusion; *PTS* Primary tumor size; *SUVmax* Maximum standard uptake value; *SUVmean* Mean standard uptake value.

The histopathological types of these patients encompassed 3 fibromas, 4 thecomas, 9 theca fibromas, and 5 granulosa cell tumors (adult type). Among the 21 patients, 15 exhibited serum Ca125 values outside the normal reference range, ranging from 135.4 to 2666. All 21 patients had unilateral primary tumors, with 8 located in the left ovary and 13 in the right ovary. The maximum diameter of the primary tumors ranged from 2.7 to 19.0 cm, with a median of 8.4 cm. With the exception of 5 patients with uniform tumor density, the remaining patients displayed uneven tumor density, including low-density cystic degeneration in 14 out of 21 patients and high-density calcification in 9 out of 21 patients. These tumors exhibited mild to moderate elevated uptake of ^18^F-FDG on PET, with SUVmax and SUVmean ranging from 2.1 to 8.6 and 0.5 to 3.0, respectively. Out of the enrolled patients, 20 presented with ascites, 15 with pleural effusion (either right or bilateral), and 14 with both ascites and pleural effusion.

**Figure 2:**
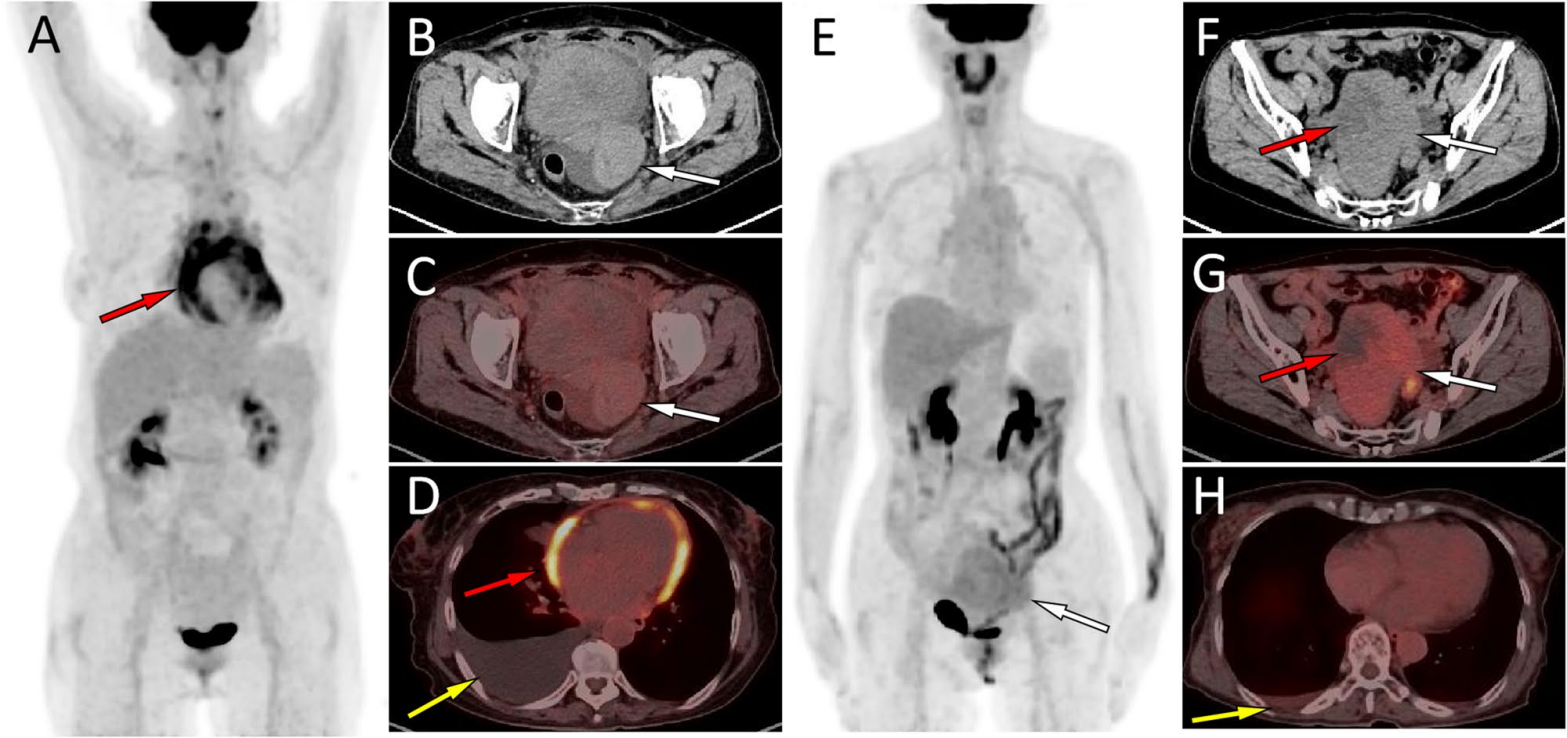
(**A**–**D**) Representative ^18^F-FDG PET/CT images of theca fibroma in a 72-year-old woman who sought medical help for two weeks because of pain in the precordial area and difficulty breathing. The maximum intensity projection (MIP, **A**) showed no significant increase in ^18^F-FDG uptake in the rest of the body except in the pericardial area (red arrow). Axial CT (**B**, arrow) showed a mass of first order soft tissue density on the left side of the pelvic cavity, and no significant uptake of ^18^F-FDG was observed on the corresponding PET/CT fusion (**C**, arrow). Axial PET/CT reveals that the lesions shown on the MIP map are pericardial effusions with increased diffuse uptake of ^18^F-FDG (**D**, red arrow). In addition, an intermediate pleural effusion without increased uptake of ^18^F-FDG was seen in the right thoracic cavity (**D**, yellow arrow). (**E**–**H**) Representative ^18^F-FDG PET/CT images of granulosa cell tumor (adults type) in a 45-year-old woman who sought medical attention for one month due to right chest pain. The MIP map shows a shadow with slightly increased ^18^F-FDG uptake in the lower abdomen (**E**, arrow). Axial CT (**F**) showed that the lesion was a soft tissue mass (white arrow) with low density cystic degeneration (red arrow) in the pelvic cavity. The axial PET/CT (**G**) of the corresponding site showed a mild increase in ^18^F-FDG uptake in the solid part (white arrow) of the tumor, with a SUVmax of 2.4, while no radioactive uptake was observed in the cystic area (red arrow). Axial PET/CT at the chest level showed a small amount of fluid in the right thoracic cavity without radioactive uptake (**H**, yellow arrow), and the amount of fluid accumulation is significantly lower than the amount of fluid accumulation in the serous cavity caused by serous cavity effusion.

**Figure 3:**
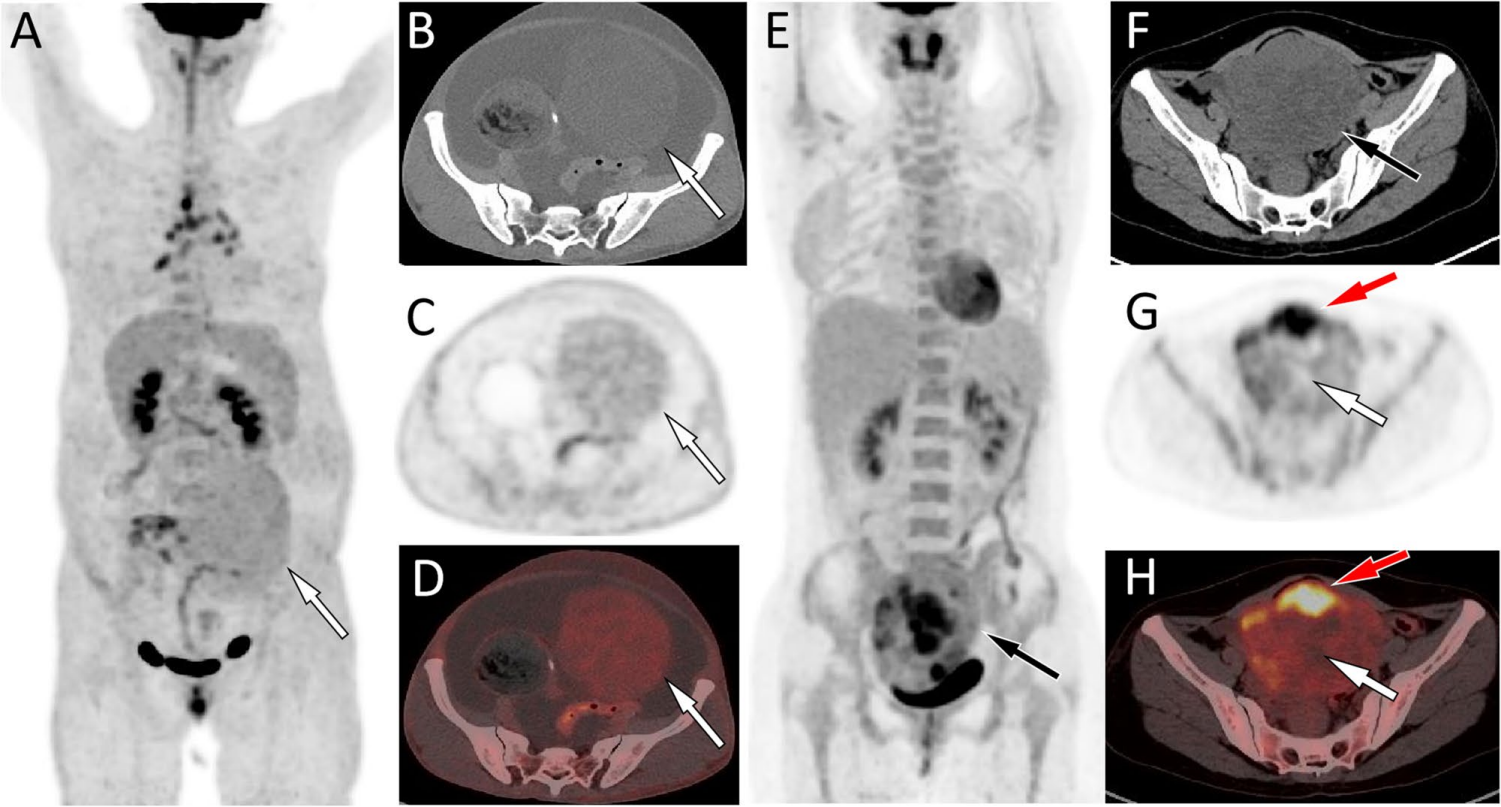
(**A**–**D**) Representative ^18^F-FDG PET/CT images of fibroma in a 59-year-old woman who admitted to hospital due to abdominal pain for 2 months. The maximum intensity projection (MIP, **A**) showed a slightly increased ^18^F-FDG uptake shadow in the lower abdomen (arrow). Axial CT of the corresponding site revealed a soft tissue mass of uniform density on the left side of the pelvic cavity (**B**, arrow). Axial PET (**C**) and PET/CT fusion (**C**) images showed a uniform and slightly increased ^18^F-FDG uptake, with a SUVmax of 2.2. (**E**–**H**) Another representative ^18^F-FDG PET/CT images of granulosa cell tumor (adults type) in a 45-year-old woman who admitted to hospital due to chest and abdominal pain for 2 weeks. The MIP (**E**) showed an increased ^18^F-FDG uptake shadow in the lower abdomen (arrow). Axial CT showed that the shadow was located in the pelvic cavity and was a soft tissue density mass (**F**, arrow). Axial PET (**G**) and PET/CT fusion (**H**, arrow) images showed varying degrees of increased uptake of ^18^F-FDG in the solid components of the tumor, with a SUVmax of 6.8 (red arrow), while the cystic area did not uptake ^18^F-FDG (white arrow). From these two representative cases, it can be seen that the SUVmax of granulosa cell tumors is significantly higher than that of fibromas.

**Figure 4:**
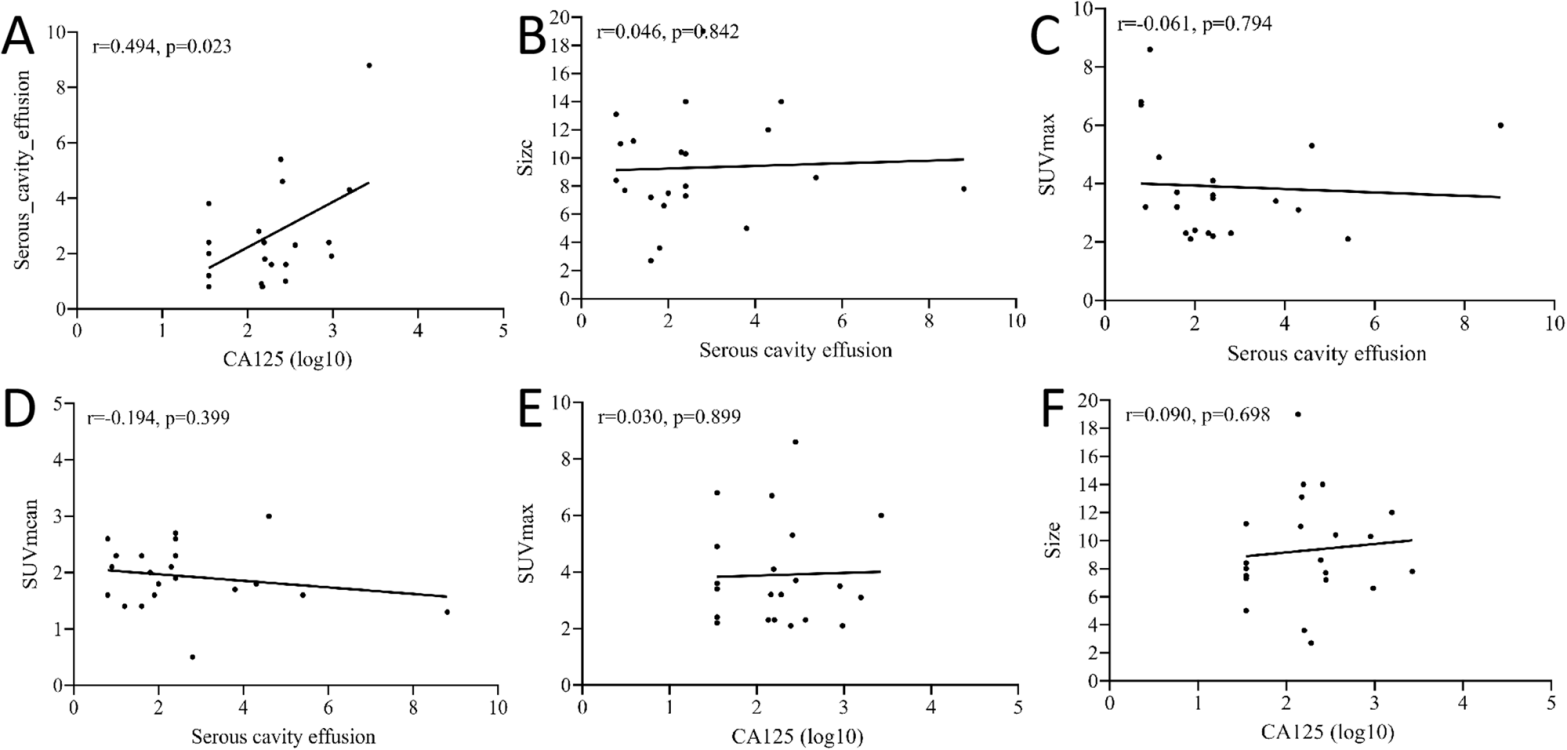
Correlation analysis showed that the amount of serous cavity effusion was positively correlated with the value of serum Ca125 (**A)**. The correlation analysis of the size of tumors versus serous cavity effusion (**B**), the SUVmax of tumors versus serous cavity effusion (**C**), the SUVmean of tumors versus serous cavity effusion (**D**), the SUVmax of tumors versus the value of serum Ca125 (**E**) and the size of tumors versus the value of serum Ca125 (**F**) showed no correlation.

Table S1 presents the clinical features and PET/CT findings of the 21 patients, categorized by their histopathological types. It is observed from the table that statistical differences were found in several different pathological types of sex cord stromal tumors concerning patient age, the volume of fluid in the serous cavity effusion, the density of the primary tumor, and SUVmax. Notably, the age at diagnosis of theca fibroma was significantly higher than that of granulosa cell tumor, and the amount of serous cavity effusion caused by the former was significantly higher than that caused by the latter (as demonstrated in Fig. 2). For the CT component of the PET/CT images, significant differences in densities were detected between granulosa cell tumors and fibromas. All granulosa cell tumors displayed inhomogeneous densities with cystic degeneration, while all fibromas exhibited homogeneous solid densities. In terms of PET, SUVmax was significantly higher for granular cell tumors than for fibromas (as shown in Fig. 3). Subsequent correlation analysis indicated a positive correlation between the volume of serous cavity effusion and the value of serum Ca125 (*P* < 0.05), as illustrated in Fig. 4. However, no relationship was identified between the serous cavity effusion volume and primary tumor size, SUVmax, or SUVmean. Additionally, serum Ca125 value showed no correlation with tumor size and SUVmax.

All patients experienced the resolution of pleural and ascitic fluid within 1–2 months after surgery, and their serum Ca125 levels returned to normal.

## Discussion

MS, a rare condition characterized by benign ovarian tumors accompanied by pleural effusion and ascites, has garnered significant attention since its initial report by Professor Meigs in 1937^9,10^. This disease predominantly affects menopausal women around the age of 50, who often present with medical concerns related to ascites and pleural effusion, including chest and abdominal discomfort, bloating, and respiratory difficulties^5^. In the current study, the average age of patients in the MS group is 54 years, and they sought medical attention primarily due to symptoms such as abdominal pain, bloating, or respiratory distress, aligning with findings in the existing literature.

Serum tumor marker analysis, including Ca125 and HE4, is a crucial aspect of ovarian tumor diagnosis, as these markers are frequently elevated in most epithelial ovarian cancer cases^11^. However, this elevation is not specific, as it can also occur in other conditions such as peritonitis, endometriosis, intestinal adenocarcinoma, and certain benign ovarian tumors, particularly when coexisting with MS^8^. The surge in serum Ca125 levels among MS patients is attributed to tumor-induced peritoneal stimulation, resulting in high Ca125 expression in peritoneal mesothelial cells, rather than tumor secretion itself. This is substantiated by negative Ca125 immunohistochemical staining in the tumors^12^. Additionally, research has established a positive correlation between serum Ca125 levels and the quantity of serous cavity effusion in MS patients^13,14^. Among the 21 MS patients included in our study, 15 exhibited elevated serum Ca125 levels, consistent with previous literature, and correlation analysis further confirmed a positive relationship between Ca125 levels and serous cavity effusion volume.

Traditional imaging methods, such as ultrasound, CT, and MRI, have been detailed in prior studies on MS^15^. Nevertheless, the PET/CT characteristics of this condition have yet to be thoroughly explored. PET/CT offers a comprehensive assessment of the overall condition, supplying precise anatomical localization and metabolic information about the lesion, which is crucial for understanding and diagnosing MS. Our current study primarily encompasses cases of MS induced by ovarian sex cord stromal tumors. Among these cases, fibromas typically exhibit a uniform solid density on CT and demonstrate mild increases in ^18^F-FDG uptake on PET, aligning with previously reported PET/CT findings for ovarian fibromas^16^. Thecomas and theca fibromas predominantly manifest as solid soft tissue density masses, but they are more prone to cystic changes and necrosis. Similar to fibromas, thecoma and theca fibromas display only a mild increase in 18F-FDG uptake on PET. Granulosa cell tumors also present as uneven density soft tissue masses on CT, often accompanied by calcification and/or cystic changes. However, in contrast to other sex cord stromal tumor types, they exhibit higher ^18^F-FDG uptake and a larger SUVmax on PET. Furthermore, serous cavity effusion attributed to granulosa cell tumors is typically less extensive than other types.

MS, a benign ovarian tumor associated with ascites and/or pleural effusion, can coincide with elevated Ca125 levels. Therefore, the primary distinction to be made is from epithelial ovarian cancer. A prior meta-analysis has confirmed the efficacy of PET/CT in distinguishing between benign and malignant ovarian masses, achieving a diagnostic accuracy of up to 95%^17^. Furthermore, a subsequent quantitative investigation evaluating benign and malignant ovarian tumors through the PET/CT parameter SUVmax demonstrated similarly robust diagnostic performance. This analysis revealed that the SUVmax of benign and malignant ovarian tumors averaged 2.2 and 9.6, respectively^18^. The findings of our present study substantiate these previous research outcomes by highlighting a significant disparity in ^18^F-FDG uptake between the MS and OC-PMS groups. The SUVmax in the OC-PMS group significantly surpasses that in the MS group, further corroborating the aforementioned research. Additionally, our study introduces another critical distinguishing feature: serous cavity effusion induced by ovarian cancer is more frequently associated with focal and diffuse ^18^F-FDG uptake. In contrast, the MS group does not exhibit such characteristics. The rationale behind this discrepancy lies in the fact that serous cavity effusion induced by ovarian cancer is typically linked with implantation metastasis, resulting in enhanced ^18^F-FDG uptake. Other benign pelvic tumors, such as ovarian teratomas and uterine leiomyomas, can also give rise to serous cavity effusion, resembling benign pseudo-MS, and may be accompanied by elevated Ca125 levels. Therefore, differentiation from MS is imperative^19^. Ovarian teratomas typically feature discernible fat and calcified components on CT scans, while uterine leiomyomas usually present comparable density and ^18^F-FDG uptake, equivalent to normal uterine parenchyma^20,21^, thereby distinguishing them from MS induced by ovarian sex cord stromal tumors.

The principal treatment for MS revolves around the surgical removal of primary ovarian tumors, with laparoscopic surgery being especially advantageous due to its minimal invasiveness, mild postoperative discomfort, and speedy recovery^14,22^. The prognosis for MS is favorable, characterized by the complete resolution of ascites and/or pleural effusion following the excision of the primary tumor. Rapid symptom relief is achieved, and the recurrence of ascites and pleural effusion is unlikely post-tumor resection^23^. Following preoperative PET/CT assessment, all patients included in our study underwent surgical mass resection, leading to the rapid disappearance of serous cavity effusion within a short timeframe, along with subsequent relief from clinical symptoms. Notably, among the patients enrolled, one exhibited pericardial effusion and notably increased ^18^F-FDG uptake on PET. Her pericardial effusion and chest discomfort persisted despite preoperative anti-inflammatory therapy but promptly subsided within one week after the surgical removal of the ovarian tumor. Ultrasound examinations revealed complete resolution of pericardial effusion, thus confirming the ovarian tumor as the underlying cause and corroborating the diagnosis of MS.

However, this study has significant limitations due to the rare nature of MS and the small number of cases. Furthermore, rigorous study design typically demands the inclusion of patients with ovarian sex cord stromal tumors lacking pleural effusion and ascites as a control group, which is another limitation. Nonetheless, given the specific nature of PET/CT examinations, only patients suspected of malignancy or those without a definitive benign or malignant diagnosis before surgery undergo further evaluation. This, in turn, leads to the collection of a limited number of cases, precluding their inclusion as controls for comparative analysis.

## Conclusion

In conclusion, our study underscores the PET/CT findings associated with MS, characterized by primary ovarian tumors exhibiting mildly increased ^18^F-FDG uptake and serous cavity effusion typically lacking ^18^F-FDG uptake in the absence of inflammation in MS patients. The volume of serous cavity effusion demonstrates no significant correlation with tumor size, SUVmax, SUVmean, and other parameters, but does exhibit a positive correlation with Ca125 levels. Familiarity with the PET/CT findings related to MS can aid in securing an accurate diagnosis prior to surgery.

### Supplementary Information

Supplementary Table S1.

## Data Availability

The authors confirm that the data supporting the findings of this study are available within the article.

## Abbreviations

CT: Computed tomography
^18^F-FDG: Fluoro-18 fluorodeoxyglucose
PET: Positron emission tomography
MRI: Magnetic resonance imaging
ROI: Region of interest
MS: Meigs syndrome
SUVmax: Maximum standardized uptake value
SUVmean: Mean standardized uptake value
OC-PMS: Pseudo-Meigs syndrome caused by ovarian cancer
