# Supplementary material for: 18F-FDG PET/CT features of Meigs syndrome induced by ovarian sex cord stromal tumors: a retrospective clinical study
Source: Sci Rep. 2024 Jan 3;14:347. doi: 10.1038/s41598-024-51186-5 (PMC10764332; doi:10.1038/s41598-024-51186-5)
Supplement: Supplementary file 1 — Supplementary Table S1. [file 41598_2024_51186_MOESM1_ESM.docx]

**Table S1**. Clinical and PET/CT features of Meigs syndrome induced by different pathologic types of sex cord stromal tumors.

| Parameters | Fibroma (N=3) | Thecoma  (N=4) | TF (N=9) | GCT (N=5) | *P* | *P1* | *P2* | *P3* | *P4* | *P5* | *P6* |
| --- | --- | --- | --- | --- | --- | --- | --- | --- | --- | --- | --- |
| Age (years) | 46.33±11.68 | 61.50±15.09 | 61.33±10.84 | 40.80±5.17 | **0.012** | 0.505 | 0.195 | 0.138 | 0.066 | **<0.001** | 0.985 |
| Ca125 (  μ/mL) | 159.00 [97.00; 259.95] | 520.70 [142.75; 912.65] | 245.00 [156.00; 280.50] | 35.00 [35.00; 148.90] | 0.354 | 0.341 | 0.48 | 0.642 | 0.135 | 0.117 | 1 |
| PTS (cm) | 7.33±3.45 | 11.72±5.22 | 8.73±3.90 | 9.58±2.46 | 0.478 | 0.391 | 0.239 | 0.589 | 0.49 | 0.629 | 0.356 |
| SCE (cm)* | 2.30 [2.05; 2.35] | 2.15 [1.65; 2.50] | 3.80 [2.40; 4.60] | 1.00 [0.80; 1.20] | **0.023** | 0.051 | 0.858 | 0.225 | 0.14 | **0.006** | 0.162 |
| Density |  |  |  |  | **<0.001** | **0.018** | 0.086 | 0.051 | 0.058 | 0.103 | 0.53 |
| CD | 0 (0.00%) | 1 (25.00%) | 1 (11.11%) | 1 (20.00%) |  |  |  |  |  |  |  |
| SD | 0 (0.00%) | 3 (75.00%) | 5 (55.56%) | 4 (80.00%) |  |  |  |  |  |  |  |
| USD | 3 (100.00%) | 0 (0.00%) | 3 (33.33%) | 0 (0.00%) |  |  |  |  |  |  |  |
| Cystic change | |  |  |  | **0.031** | **0.018** | 0.143 | 0.182 | 0.444 | 0.258 | 1 |
| no | 3 (100.00%) | 1 (25.00%) | 3 (33.33%) | 0 (0.00%) |  |  |  |  |  |  |  |
| yes | 0 (0.00%) | 3 (75.00%) | 6 (66.67%) | 5 (100.00%) |  |  |  |  |  |  |  |
| Calcification | |  |  |  | 0.055 | 0.143 | 0.143 | 1 | 1 | 0.091 | 0.217 |
| no | 3 (100.00%) | 1 (25.00%) | 7 (77.78%) | 3 (60.00%) |  |  |  |  |  |  |  |
| yes | 0 (0.00%) | 3 (75.00%) | 2 (22.22%) | 2 (40.00%) |  |  |  |  |  |  |  |
| SUVmax | 2.30 [2.25; 2.30] | 2.75 [2.25; 3.28] | 3.60 [3.20; 4.10] | 6.70 [4.90; 6.80] | **0.024** | **0.024** | 0.463 | 0.052 | 0.05 | 0.096 | 0.122 |
| SUVmean | 2.00±0.10 | 1.62±0.81 | 2.04±0.62 | 1.94±0.50 | 0.704 | 0.806 | 0.422 | 0.84 | 0.526 | 0.738 | 0.4 |

Notes:*Diameter at the thickest point; PTS, primary tumor size; SCE, serous cavity effusion; CD, cystic dominance; SD, solid dominance; USD, uniform solid density; GCT, granulosa cell tumor; TF, theca fibroma; *P*, overall; *P1*, fibroma vs GCT; *P2*, fibroma vs TCT; *P3*, fibroma vs TF; *P4*, GCT vs TCT; *P5*, GCT vs TF; *P6*, TCT vs TF; ****P* < 0.001.
